# The Post-Coronavirus World in the International Tourism Industry: Application of the Theory of Planned Behavior to Safer Destination Choices in the Case of US Outbound Tourism

**DOI:** 10.3390/ijerph17186485

**Published:** 2020-09-06

**Authors:** Heesup Han, Amr Al-Ansi, Bee-Lia Chua, Beenish Tariq, Aleksandar Radic, Su-hyun Park

**Affiliations:** 1 College of Hospitality and Tourism Management, Sejong University, 98 Gunja-Dong, Gwanjin-Gu, Seoul 143-747, Korea; heesup.han@gmail.com (H.H.); amralansi1@gmail.com (A.A.-A.); 2 Department of Food Service and Management, Faculty of Food Science and Technology, Universiti Putra Malaysia, Serdang 43400, Selangor, Malaysia; beelia.chua@gmail.com; 3 NUST Business School, National University of Sciences and Technology, Sector H-12, Islamabad 44000, Pakistan; beenish.tariq@nbs.nust.edu.pk; 4 Independent Researcher, Gornji kono 8, 20000 Dubrovnik, Croatia; 5 Department of Hotel and Tourism, Incheon Jaeneung University, 178, Jaeneung-ro, Dong-gu, Incheon 22574, Korea; kayla9252@gmail.com

**Keywords:** post-COVID-19, theory of planned behavior (TPB), health belief, perceived risk, protection plan, monetary promotion, approach behavior, US international travelers

## Abstract

The tourism industry has been seriously suffering from the coronavirus disease (COVID-19) crisis ever since its outbreak. Given this pandemic situation, the major aim of this study is to develop a conceptual framework that clearly explains the US international tourists’ post-pandemic travel behaviors by expanding the theory of planned behavior (TPB). By utilizing a quantitative process, the TPB was successfully broadened by incorporating the travelers’ perceived knowledge of COVID-19, and it has been deepened by integrating the psychological risk. Our theoretical framework sufficiently accounted for the US tourists’ post-pandemic travel intentions for safer international destinations. In addition, the perceived knowledge of COVID-19 contributed to boosting the prediction power for the intentions. The associations among the subjective norm, the attitude, and the intentions are under the significant influence of the tourists’ psychological risks regarding international traveling. The comparative criticality of the subjective norm is found. Overall, the findings of this study considerably enhanced our understanding of US overseas tourists’ post-pandemic travel decision-making processes and behaviors.

## 1. Introduction

Since its earliest case was detected in December 2019, the coronavirus disease, which is formally known as COVID-19, has rapidly spread around the world [1]. It is estimated that COVID-19 has affected more than 213 countries/regions across the globe [2]. As of 25 May 2020, there have been more than 5,206,614 confirmed cases of COVID-19 globally, which includes 337,736 deaths [3]. Every country has been making tremendous efforts to limit the further spread of COVID-19 and minimize the number of cases and deaths. Irrefutably, COVID-19 has had a considerable impact on the entire economy of the world. Every country is suffering economic injuries that are derived from COVID-19. Almost all businesses/industries are under the devastating effects of COVID-19. That being said, the tourism sector is one of the hardest-hit industries because of the ongoing pandemic [4,5,6]. The global tourism industry has been facing its most serious crisis in history [5], ever since COVID-19 was declared as a pandemic by the World Health Organization (WHO) on 11 March 2020. The impact of COVID-19 on the US tourism industry is especially serious compared to other countries around the world. The US infections hit a global high, which passed every country in Europe and Asia. As of 25 May 2020, the total number of confirmed cases from the US were more than 1,568,448, which is the highest number around the world [3]. It is apparent that COVID-19 has fatally shrunk the international tourism industry ever since its outbreak, which has significantly reduced the overall tourism industry revenue [4,6] and has changed travel behaviors [1]. 

Under this uncertain tourism world, studying and understanding overseas travelers’ post-pandemic behaviors is of the utmost criticality for every destination practitioner and researcher in the international tourism industry. In particular, US international travelers’ post-pandemic destination choice processes/behaviors remain unpredictable, even though the US is the country that is the most seriously affected by COVID-19. This research therefore focuses on the post-pandemic safer destination choice behaviors among US international travelers, since no research has yet investigated and predicted their post-pandemic safer destination selection process. Many researchers in tourism, social psychology, and consumer behavior agree that investigating the underlying factors that lead to travelers’ particular intentions/behaviors, which consider volitional (social and attitudinal) and nonvolitional constituents, provides clear insights into travelers’ decision-making process for a destination/product/service/brand [7,8,9,10,11,12]. Accordingly, the conceptual framework from the present research is derived from the theory of planned behavior (TPB) [13,14]. These types of volitional and nonvolitional dimensions are the key components of the TPB [15,16,17]. This sociopsychological theory has a strong prediction power for a broad range of traveler behaviors, which assist researchers and practitioners to better understand the traveler decision formations for these types of behaviors [14,15,18].

Undeniably, the TPB has been proven to be helpful to explain travelers’ decision-making processes. Nonetheless, its sufficiency has frequently been questioned [8,19]. In addition, little empirical research has expanded the TPB for a more comprehensive explication of travelers’ choice decisions/behaviors for safe tourism destinations/products, despite the theory’s high applicability. Moreover, considerable evidence in the recent literature indicated the essential role of the perceived knowledge and the psychological risks with increasing our understanding of customers’ safe/risky behavioral choices [16,20,21,22]. However, no research has yet broadened the TPB by comprising the interplay between the key factors of this theory and the perceived knowledge and deepened the TPB by encompassing the possible influence of the psychological risk into the theory. Undoubtedly, the international tourism industry cannot be the same as before after the pandemic, even though COVID-19 is completely under control. The international tourism and traveler behaviors around the globe remain unclear [4]. Under this uncertain global tourism marketplace after the pandemic, knowing US overseas travelers’ safer destination choice mechanisms is now more important than ever before. 

The main purpose of the present research is to build a theoretical framework that explicates US overseas tourists’ post-pandemic international tourism decision formations for safer destinations by applying the TPB. Specifically, we aimed (1) to extend the TPB by integrating US overseas travelers’ perceived knowledge of COVID-19, (2) to deepen the TPB by taking into account the influence of US travelers’ psychological risks that are pertinent to international tourism into account, (3) to uncover the mediating effect of the research constructs within the proposed conceptual framework, and (4) to assess the relative importance among the study variables to determine US travelers’ post-pandemic tourism behaviors for safer destinations. Consequently, a multivariate data assessment and process is established to achieve the study objectives.

## 2. Literature Review

### 2.1. Theory of Planned Behavior and Its Focal Constituents

The TPB is one of the most broadly used sociopsychological theories designed to predict human decisions and behaviors [16,21]. Its applicability and anticipation power for diverse human behaviors have been demonstrated through meta-analyses [23]. Undeniably, the TPB is also a commonly applied theory in the tourism domain to explicate travelers’ decision-making processes and behaviors [10,12,24]. The important aspect of these types of decision formations and behaviors comprise the tourism destination choices [12,14]. Thus, the utilization of the TPB is often considered to be efficient in a wide range of destination selection processes and behaviors [8,21,24]. Under the framework of the TPB, individuals’ intentions/behaviors can be sturdily explained because of its scope, which comprises volitional and nonvolitional processes [7,10,21].

The components of the TPB are the attitude toward the behavior, the subjective norm, and the perceived behavioral control [7,17]. The attitude toward the behavior and the subjective norm are the constituents of the volitional process, whereas the perceived behavioral control is the major factor of the nonvolitional process [13,19]. The TPB framework posits that the behavioral intention is the most proximal determinant of the actual behavior [14,16] and that this intention is built based on the attitude toward the behavior, the subjective norm, and the perceived behavioral control [21]. In other words, the individuals’ intention develops through volitional and nonvolitional procedures in a simultaneous manner [16,24]. The TPB is an advanced model of the theory of reasoned action (TRA) [7,13]. Unlike the TPB, the TRA solely considers the volitional dimension to predict human behaviors [7,17,21].

The attitude toward the behavior is undoubtedly a salient determinant of travelers’ intentions/decisions [10,21,25]. This concept indicates individuals’ general assessments regarding whether a particular behavior is either positively or negatively valued [7,19]. The subjective norm is another critical predictor of travelers’ behavioral intentions [12,24]. The subjective norm refers to an individual’s perception of social pressure to or not to perform a particular behavior [16,17]. The perceived behavioral control is also a crucial determinant of the traveler intention [10]. This nonvolitional factor indicates an individual’s perception of their capability to or not to be involved in a particular behavior [19]. The positive associations among the attitude, the subjective norm, the perceived behavioral control, and the behavioral intention have long been tested and demonstrated in the extant studies of tourism and consumer behavior [9,10,12]. These studies empirically supported the conceptual justification of the TPB by Ajzen [13], which the customer’s behavioral intention for a specific action develops based on the influence of a positive attitude toward the action, the perceived social pressure, and the perceived ability to carry out the action.

### 2.2. Expanding the Theory of Planned Behavior

Even though the competence of the TPB has been proven in diverse settings, the findings in previous studies indicated that its anticipation ability for decisions/behaviors still needs an enhancement through expanding its framework [10,19,24]. In particular, the TPB overlooked the effect of the perceived knowledge and the psychological risk that are considered to be crucial to explicate individuals’ purchase decision-making processes, especially for safe/risky products. Many studies in the extant literature, particularly in tourism, have shown that the perceived knowledge and the psychological risk are critical concepts to clearly understanding the customers’ decision formations and behaviors for reliable/uncertain tourism products [20,25,26,27,28]. In addition, the fundamental role of these concepts with forming the intention has been demonstrated in abundant researches about consumer behaviors and social psychology [28,29,30,31]. The empirical cues in these studies imply that the individual’s perceived knowledge and the psychological risk could be efficiently incorporated into a rational choice theory. 

The integration of these essential concepts could enhance the theory’s ability to predict the customers’ intentions/behaviors, especially when these types of intentions/behaviors are not completely accounted for by the volitional and nonvolitional processes. Based on this, our conceptual framework provided a sound rationale regarding the combined consideration of the role of the perceived knowledge, the psychological risk, and the original TPB constituents into one sturdy framework. In sum, the theoretical premise of this research in the international tourism sector is that the travelers who have strong knowledge of COVID-19 are likely to form a positive attitude toward safer destination choice behaviors and to perceive the social norm to practice the action, which leads to the increased intention to engage in the behavior in conjunction with the perceived behavioral control. Moreover, the chain of the subjective norm, the attitude toward the behavior, and the intention relationships are strengthened by the psychological risk related to overseas tourism.

### 2.3. Perceived Knowledge and Its Role

Travelers’ perceived knowledge and the serious concern of the safety/social/environmental issues of tourism destinations have long been important concepts to explain their behaviors [29,30,32]. The perceived knowledge, which is a cognitive variable, indeed plays a crucial role in tourists’ product/brand/destination choices in the international tourism industry [30,33,34]. In the tourism sector, the perceived knowledge indicates one’s ability to know and understand a variety of tourism-related issues, problems, and behaviors [33]. Travelers tend to avoid a situation where their knowledge to direct their specific actions is not sufficient [30]. In other words, travelers are likely to reduce the possible uncertainty by not practicing the action when their knowledge is not enough to guide a certain type of action. Individuals often think their perceived level of knowledge about an object/product/event/issue is high when they believe that they know/understand it better compared to others [33]. The existing empirical studies indicated that travelers’ perceived knowledge as a critical cognitive factor is an important determinant of the attitudinal and the social variables in their decision formations and behaviors [29,30,33]. In-line with the evidence from these studies, tourists’ perceived knowledge of COVID-19 can be the essential driver of their subjective norms and attitudes to generate an approachable decision for safer international tourism destination.

### 2.4. Relationship between the Subjective Norm and the Attitude toward the Behavior

The essential role of the attitude toward the behavior and the subjective norm as predictors of intention has been well-documented [10,12,24]. Yet, many studies asserted the strong need of the refinement of the existing sociopsychology theory by adding/altering the path(s) between these variables [35,36,37,38]. These studies demonstrated that inserting a causal relationship between the normative factor and the attitudinal factor within the TPB makes the framework more parsimonious, which eventually fortifies the theory. Indeed, Han et al. [37] attempted to link the subjective norm and the attitude toward the behavior for a hospitality product. Their findings showed that the individuals’ perceived social pressure from critical others contributed to increasing their attitudes toward the specific behaviors. They empirically verified that the prediction power of the TPB was significantly enhanced when the linkage was added. In the hotel sector, Han and Kim [36] explored the guests’ intention generation processes. Their results revealed that the extended TPB framework, which comprises the linkage from the social dimension and the attitudinal dimension, better accounted for the total variance in the guests’ behavioral intentions for a hotel product than the original TPB.

### 2.5. Psychological Risk and Its Influence

For the past few decades, the concept of the perceived risk has long been the critical subject of diverse research [28,31,39]. Particularly, the perceived risk in the tourism literature is quite extensive [26,28,31,40,41]. The psychological risk is the key facet of the perceived risk, whose nature is multidimensional [20,39,42]. The important aspect of the psychological risks in tourism is the travel concern [28]. The psychological risks embracing this concern include fear, unnecessary tension, anxiety, and discomfort, which are related to traveling among tourists as its constituents [20,28,31]. The term psychological risk in tourism refers to the risk of anxiety/stress/discomfort/fear that stems from being a tourist [39].

A considerable amount of the existing literature has unearthed the possible influence of the risk perception on individuals’ decision-making processes and behaviors [20,26,28]. Particularly, in the tourism sector, the possibility of the fatal incidents while traveling boosts tourists’ psychological risk perceptions [39]. When individuals feel concerned about the possibility of the occurrence of failure while traveling, they often avoid tourism activity or postpone their tourism plans [26,39], which means that individuals’ tourism decision formations and behaviors are largely influenced by their psychological risks [26,28]. Undoubtedly, this risk perception is also of significance in travelers’ international destination choice behaviors. Indeed, the psychological risk and its role have been extensively researched in many destination studies [20,28,31,40,43]. 

For instance, in the Muslim tourism sector, Al-Ansi et al. [20] explored that tourists’ general risk perceptions, which were comprised of psychological risks, affect the formation of international Muslim travelers’ behavioral intentions. Law [26] unearthed overseas tourists’ travel decision-making processes for international tourism destinations where there exists the probability of the occurrences of infectious diseases, terrorism, or disasters. His empirical findings revealed that tourists’ international travel decision formations are under the considerable impact of their perceived levels of risk. The important moderating nature of risk perception has been also asserted by Han et al. [41]. Their recent research demonstrated the effect of travelers’ psychological risks related to inconveniences at international tourism destinations on the relationships among the cognitive factors, the affective factors, and the motivational factors and the behavioral intentions. In all, these studies discussed above indicated that the travelers’ risk perceptions as a moderator influence the relationship strength between travel decisions and their drivers.

### 2.6. The Proposed Model and the Research Hypotheses

**Figure 1:**
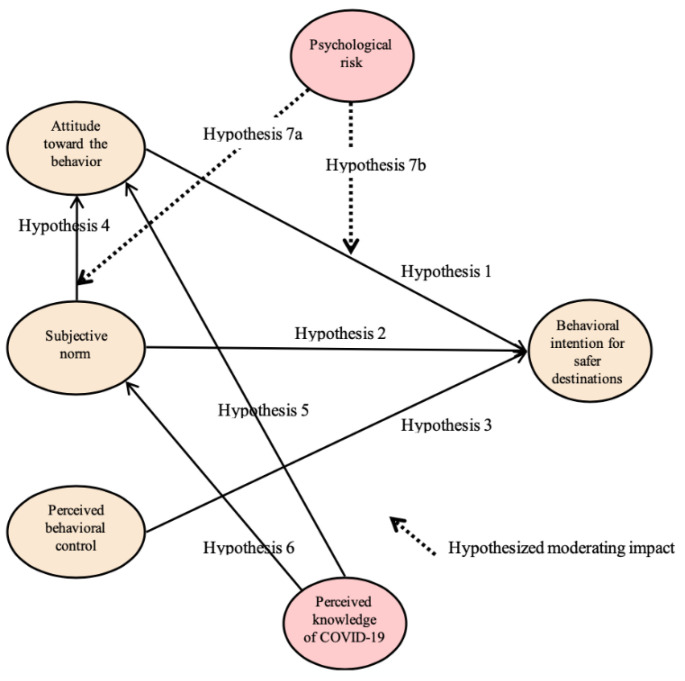
Proposed theoretical model. H1: The attitude toward the behavior has a positive impact on the behavioral intentions for safer destinations among US international travelers. H2: The subjective norm has a positive impact on the behavioral intentions for safer destinations among US international travelers. H3: The perceived behavioral control has a positive impact on the behavioral intentions for safer destinations among US international travelers. H4: The subjective norm has a positive impact on the attitude toward behaviors among US international travelers. H5: The perceived knowledge of COVID-19 has a positive impact on the attitude toward behaviors among US international travelers. H6: The perceived knowledge of COVID-19 has a positive impact on the subjective norm among US international travelers. H7a: The psychological risk significantly moderates the relationship between the subjective norm and the attitude toward behaviors among US international travelers. H7b: The psychological risk significantly moderates the relationship between the attitude toward behaviors and behavioral intentions for safer destinations among US international travelers.

The proposed conceptual model is displayed in Figure 1. The model is comprised of six research variables and seven hypotheses. Within the proposed theoretical framework, the psychological risk is included as a moderator. Hypotheses 1, 2, 3, 4, 5, and 6 are related to the causal relationships among the theoretical constructs to generate the travelers’ behavioral intentions for safer destinations. Meanwhile, Hypothesis 7a and Hypothesis 7b are related to the moderating role of the psychological risk.

## 3. Methods

### 3.1. Measures

The survey questionnaire included a description of the research, the measures for the study variables, and questions for the personal demographic information. All the measures for the research constructs are adopted from the extant social psychology and consumer behavior studies [8,13,19,28,31,33,44]. All the study constructs were evaluated with multiple items. In particular, three items for the attitude, three items for the subjective norm, three items for the perceived behavioral control, and three items for the behavioral intention for safer destinations were used. In addition, we utilized three items for the perceived knowledge of COVID-19 and three items for the psychological risk. The survey questionnaire containing these measures was pretested with tourism academics. A slight amendment was made based on their feedback. Moreover, three academic experts reviewed and improved the survey questionnaire. All the measurement items used in this research are displayed in the Appendix A.

### 3.2. Data Collection Procedure

The study employed an online survey method to collect the data. The developed survey questionnaire was sent to general US international tourists who have experienced overseas traveling to a country located in any different continent other than North America at least once in the past three years. The rationale of selecting the sampling from the US is due to travel restrictions enforced in many affected destinations across Europe and Asia, which are most visited outbound destinations by US international tourists [5,6]. Even though the US was lately affected by the pandemic outbreak, many US cities were in the early stage of infection of COVID-19 compared to other places in Europe (e.g., Italy) and Asia (e.g., China) [3,5]. The samples were chosen through an online survey company’s customers information and database in a random manner. The survey invitation email was delivered to the potential survey participants. They were requested to click the URL within the invitation email, which led to the survey. When accessing the survey, all respondents were invited to thoroughly check and read the research description and the survey instructions, which involved reliable sources and updated information reported by the WHO. Only those who were older than 18 years old were asked to fill out the survey. About 1300 survey invitations were sent out in the middle of April, 2020 in the USA. A prior screening question was asked (i.e., Have you visited any destination in Europe or Asia within the last 3 years?) to ensure valid respondents were involved. A total of 305 usable responses for our research were eventually gathered through this collection process. This sample size was sufficient to conduct a multivariate data analysis [45]. These cases were finally utilized in the analysis process. The average time that the survey participants spent to complete the questionnaire was about 15 minutes.

### 3.3. Sample Characteristics

Of the 305 survey participants, 51.5% (*n* = 157) were males and 48.5% (*n* = 148) were females. The average age was 38.87 years old. The participants’ ages ranged from 18 years old to 77 years old. In regard to the respondents’ highest education level, about 80.3% reported they were university graduates or more, which was followed by two-year college/community college graduates (11.8%), and high school graduates or less (7.9%). All the participants were US citizens. When their marital status was asked, about 66.2% indicated that they were married, which was followed by single (30.2%), and other (3.6%). In regards to their ethnic backgrounds, the majority of the respondents were Caucasian/White (79.3%), which was followed by Asian (9.8%), African American (6.2%), Hispanic (3.6%), and other (1.0%). All the participants indicated that they had visited a country in a different continent other than North America. About 53.8% reported that their most recent visit to the country in a different continent was within the last 6 months, which was followed by within 1 to 2 years (34.1%), within a month (7.2%), and within 2 to 3 years (4.9%).

### 3.4. Ethical Statement

Due to the observational nature of the study, and in the absence of any involvement of therapeutic medication, no formal approval of the Institutional Review Board of the local Ethics Committee was required. Nonetheless, all subjects were informed about the study, and participation was fully on a voluntary basis. The study was conducted in accordance with the Helsinki Declaration.

## 4. Results

### 4.1. The Measurement Model Estimation

Prior to the evaluation of the structural equation modeling, we conducted a confirmatory factor analysis, and a maximum likelihood estimation approach was employed. The results showed that the measurement model, which included all the measures for the study variables, contained an adequate level of the goodness-of-fit statistics (χ^2^ = 189.418, df = 117, *p* < 0.001, χ^2^/df = 1.619, RMSEA = 0.045, CFI = 0.976, IFI = 0.976, and TLI = 0.968). An internal consistency of the measurement items for each latent variable was evaluated. As shown in Table 1, the composite reliability values are all greater than the minimum threshold of 0.700 [45]. This result implies that the construct measures contain an appropriate internal consistency level. The average variance extracted (AVE) values were estimated. As reported in Table 1, the AVE values are all greater than Hair et al.’s [45] suggested cutoff of 0.500. The values fell between 0.533 and 0.839. These results imply that the construct measures have an acceptable level of convergent validity. The AVE values were then compared to the correlations (squired) between our theoretical constructs. All the AVE values were found to exceed the squared correlations. Therefore, discriminant validity of the construct measures is evident [46].

### 4.2. The Structural Model Estimation

**Figure 2:**
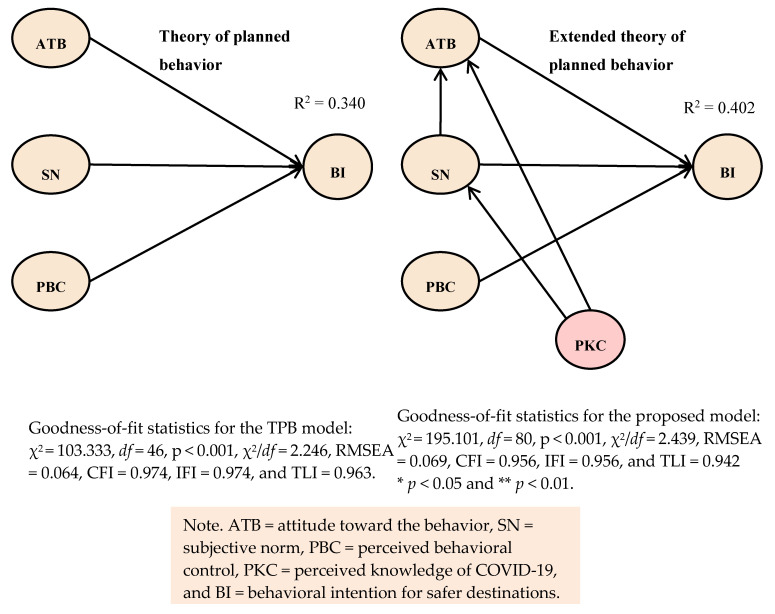
Comparison between the theory of planned behavior (TPB) and the proposed extended TPB model.

The proposed extended TPB model was estimated to evaluate the hypothesized relationships among the research variables and to assess the anticipation ability of the hypothesized theoretical framework. To accomplish this, we utilized structural equation modeling with a maximum likelihood estimation method. Our findings reported that the model satisfactorily fit the data (χ^2^ = 195.101, df = 80, *p* < 0.001, χ^2^/df = 2.439, RMSEA = 0.069, CFI = 0.956, IFI = 0.956, and TLI = 0.942). The model contained a sufficient level of anticipation power for US international travelers’ behavioral intentions for safer destinations (R^2^ = 0.402). This model was then compared to the original TPB model. As illustrated in Figure 2, the TPB model also has an adequate model fit to the data (χ^2^ = 103.333, df = 46, *p* < 0.001, χ^2^/df = 2.246, RMSEA = 0.064, CFI = 0.974, IFI = 0.974, and TLI = 0.963). However, its prediction power for behavioral intention (R^2^ = 0.340) is lower than the proposed extended TPB. While our structural model explained about 40.2% of the total variance in the behavioral intention, the original TPB model accounted for about 34.0% of the variance. This result indicated the superior ability of the proposed model as compared to the TPB model.

**Figure 3:**
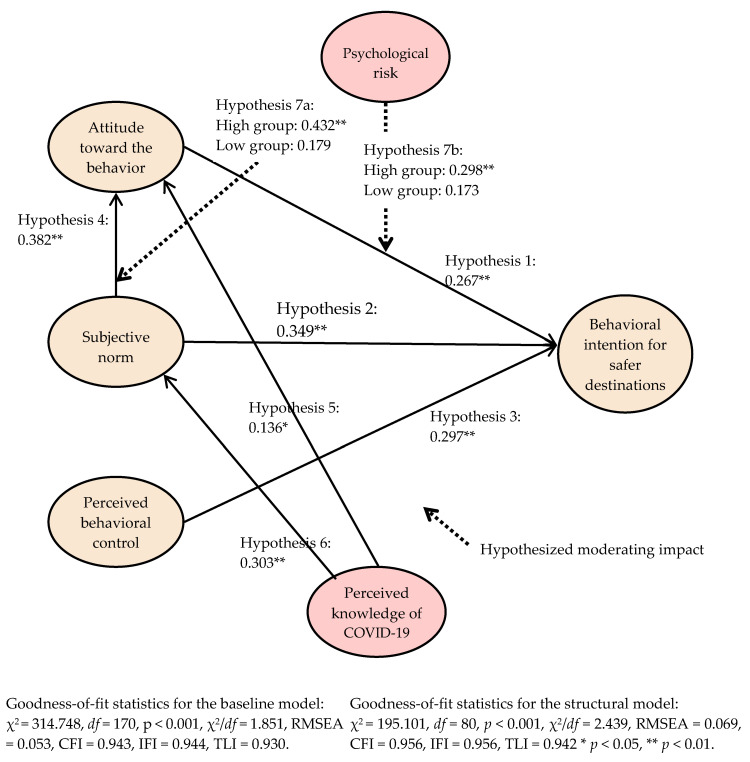
Proposed model evaluation results.

The details about the proposed model evaluation results are reported in Table 2 and Figure 3. The hypothesized paths’ impacts of the attitudes toward the behavior, the subjective norm, and the perceived behavioral control on the intention were assessed. Our results indicated that the attitude (β = 0.267 and *p* < 0.01), the subjective norm (β = 0.349 and *p* < 0.01), and the perceived behavioral control (β = 0.297 and *p* < 0.01) significantly influence the behavioral intention for safer destinations. Therefore, Hypotheses 1, 2, and 3 are supported. The linkage between the subjective norm and the attitude toward the behavior was examined. Our findings show that this link is positive and significant (β = 0.382 and *p* < 0.01). Thus, Hypothesis 4 is supported. The proposed influence of the perceived knowledge of COVID-19 was assessed. The results showed that the perceived knowledge exerted a significant impact on the attitude toward the behavior (β = 0.136 and *p* < 0.05) and the subjective norm (β = 0.303 and *p* < 0.01). Therefore, Hypothesis 5 and Hypothesis 6 are supported.

The indirect and the total effects of the study variables were estimated. Our close examination revealed that the subjective norm had a significant influence on the behavioral intention for safer destinations indirectly through the attitude toward the behavior (β = 0.102 and *p* < 0.05). In addition, the perceived knowledge of COVID-19 had a significant indirect influence on the behavioral intention (β = 0.173 and *p* < 0.01). This result implies that the attitude toward the behavior and the subjective norm acted as significant mediators within the proposed conceptual framework. Next, the total impact of the study constructs was examined. Our finding showed that the subjective norm had the greatest total influence on the behavioral intention for safer destinations (β = 0.451 and *p* < 0.01), which was followed by the perceived behavioral control (β = 0.297 and *p* < 0.01), the attitude toward the behavior (β = 0.267 and *p* < 0.01), and the perceived knowledge of COVID-19 (β = 0.173 and *p* < 0.01). This means that the subjective norm is the strongest contributor to increase US international travelers’ behavioral intentions to choose safer destinations.

### 4.3. The Baseline Model Estimation

To test the moderating effect of the psychological risk, a test for the metric invariance was conducted. First, the survey participants’ responses were split into a high group and a low group in regards to psychological risk by conducting a K-means cluster analysis. The high group included 209 cases, and the low group contained 96 cases. A baseline model that encompassed these high and low groups of psychological risk was then generated. As shown in Table 3 and Figure 3, our results demonstrated that the baseline model involved a satisfactorily level of the goodness-of-fit statistics (χ^2^ = 314.748, df = 170, *p* < 0.001, χ^2^/df = 1.851, RMSEA = 0.053, CFI = 0.943, IFI = 0.944, and TLI = 0.930). This model is used to generate the invariance models where a particular path of interest is equally constrained for the test of the hypothesized moderating effect. The details about the baseline model assessment and the invariance test results are exhibited in Table 3.

The baseline model was compared to the nested model, where the subjective norm and the attitude linkage are restricted to be equal. Our findings show that the path is significantly different across the high psychological risk group and the low psychological risk group (Δχ^2^ [1] = 5.652 and *p* < 0.05). This result supports Hypothesis 7a. The relationship strength is stronger in the high group than in the low group. However, our findings showed that the path from the attitude toward the behavior to the behavioral intention is not significantly different between the high psychological risk group and the low psychological risk group (Δχ^2^ [1] = 0.408 and *p* > 0.05). Therefore, Hypothesis 7b is not supported.

## 5. Discussions

Under the worldwide COVID-19 pandemic situation, this study focused on international tourism behaviors. Our theoretical framework is built on the TPB to provide a clear comprehension of US overseas travelers’ post-pandemic tourism decision-making processes for safer destinations, which are comparatively less affected by COVID-19. The proposed model satisfactorily expanded the existing sociopsychological theory by taking the impact of the perceived knowledge and the psychological risk into account. The proposed theoretical framework showed how the cognitive dimension, the volitional dimension, and the nonvolitional dimension drive travelers’ international tourism intentions for safer destinations. The constructs within the proposed model explained about 40.2% of the total variance with the intentions. This value was greater than that of the original TPB, which accounted for about 34.0% of the variance in intentions. When taken together, the efficacy of our theoretical framework to understand US travelers’ post-pandemic decision formations was evident.

Our findings indicated that the relationships between the perceived knowledge of COVID-19 and the focal variables within the TPB were significant. This result implies that US travelers’ perceived knowledge of COVID-19 contributed to fortifying the power of the existing sociopsychological theory when predicting their post-pandemic travel intentions for safer international tourism destinations. Even though considerable efforts were made regarding travelers’ destination choice behaviors [14,20,26,47,48,49], the criticality of travelers’ perceived knowledge of COVID-19 and its effects on their safer destination choices have been rarely uncovered. This research is, hence, meaningful with both theoretical and practical manners, because our results provided critical information about the essential role of travelers’ knowledge/awareness of the particular disease to elucidate their intention generation process for international destination choices. The present study satisfactorily added the vital dimension to the traveler behavioral intention formation, which was absent in the extant sociopsychological theory. 

Our investigation of the relative importance of the research variables demonstrated that the subjective norm better contributes to induce US travelers’ behavioral intentions as compared to other research constructs. In particular, this volitional factor acted as a prominent antecedent of post-pandemic behavioral intentions for safer destination choices. This result supported the previous research [16,21,22], which indicated the importance of the subjective norm to explain one’s safe/risk behaviors. This finding provided us valuable information that individuals’ perceived social pressures from family/friends/others are of the utmost criticality when they form any decision related to making a safe behavioral choice or taking risks. From the theoretical aspect, our findings indicated the necessity of the inclusion of this social dimension into the research framework regarding the safe choices of travelers’ or travelers’ risk-taking behaviors. From the practical perspective, for US government/tourism officials, an effective tactic to boost their citizens’ post-pandemic safer international destination choice behaviors is dealing with their subjective norms.

Evidence of the dissimilarity on travelers’ decisions for safe destination choice behaviors across the high psychological risk group and the low psychological risk group has scarcely been provided. In this research, US international tourists’ psychological risks related to overseas traveling were significantly explored to be the significant moderator in the relationship between the subjective norm and the attitude toward the behavior. The association strength was greater in the high psychological risk group (β = 0.432 and *p* < 0.01) than in the low group (β = 0.179 and *p* > 0.05). This finding implies that, at a similar level of perceived social pressure from important others, US tourists who feel a high psychological risk of traveling to any country seriously affected by COVID-19 are more likely to have a positive attitude toward safer destination choices than those with a low psychological risk. This result informs researchers and the practitioners that taking psychological risk into account when building a conceptual framework for individuals’ post-pandemic international destination selection process is a fundamental requisite. In the current global tourism marketplace that is highly uncertain, knowing travelers’ destination choice behaviors is of importance. This research successfully enriched the extant literature further, which helps researchers and practitioners to apparently understand travelers’ intricate decision-making process to engage in safer destination choices by demonstrating the effect of the psychological risks related to international tourism on this type of process.

In the present research, our proposition regarding the dissimilarity of the relationship between the attitude toward the behavior and the behavioral intention for safer destinations across the high psychological risk group and the low psychological risk group was not supported. Nonetheless, the linkage was interestingly only statistically significant in the high psychological risk group but not in the low group (high group: β = 0.298 and *p* < 0.01 vs. the low group: β = 0.173 and *p* > 0.05). Therefore, the difference on this linkage between the two groups should be meaningfully interpreted, despite the insignificant chi-square test results. These results offer tourism researchers and practitioners crucial information that US international tourists’ attitudes elicit their safer destination choice behaviors only when tourists believe that traveling to tourist destinations in countries seriously affected by COVID-19 outbreak are insecure.

According to the results of the present research, the attitude toward the behavior and the subjective norm were all crucial mediators. This finding demonstrated the mediation mechanism among the perceived knowledge, the volitional factors, and the behavioral intention within the proposed conceptual framework. The essential mediating effect of the attitude toward the behavior and the subjective norm must not be underestimated, since the results of this study confirmed the efficacy of utilizing these mediators when broadening an extant sociopsychology model. Being aware of the critical contribution of the attitude toward the behavior and the subjective norm, US government/tourism officials should deal with these factors to maximize the role of the perceived knowledge of COVID-19 to induce US travelers’ decisions to choose safer international tourism destinations for their vacation trips.

This study included a few limitations that offer the opportunity for future research. First, the present research used US international travelers’ responses as samples. Irrefutably, US travelers’ destination choices can differ from those who are from other countries located in different continents, and generalizing our findings to every international tourism behavior needs some caution. Future research should include survey participants from diverse countries for the enhancement of the external validity. Second, like many theories in social psychology, the key aspect of this research centered on the cognitive-centered view. However, some recent studies indicated the crucial role of emotional factors in traveler behaviors [19,24]. Future research needs to further extend the proposed theoretical framework by involving the emotional dimension for a more comprehensive prediction of international traveler decisions/behaviors.

## 6. Conclusions

In conclusion, we built a robust theoretical framework for US tourists’ post-pandemic travel intentions for safer international tourism destinations, which linked the perceived knowledge of COVID-19 to the focal constructs of the TPB and encompassed the moderating influence of the psychological risk through the empirical approach. By filling the essential requisites of the theory extension [7], this study effectually involved the concepts that are crucial in the international tourism and safety behavior contexts in the proposed model. The incorporated concepts also conceptually differed from the main constructs within the sociopsychological theory employed in the present study. The theoretical framework built in this research improved the prediction ability of the TPB, and it is broadly applicable in diverse tourism sectors, especially when explicating traveler decision-making processes for safe destinations/product choices. The proposed theoretical framework added important knowledge in the extant literature regarding the aspects of disease outbreak and travel behaviors, which are barely considered in a single sociopsychological theory. Overall, our theoretical framework, which is comprised of high effectiveness and applicability, is a critical tool for a better comprehension of the individual’s convoluted post-pandemic decision-making process and behavior for safer destination choices. The value of the present research both theoretically and practically is accordingly notable.

## Figures and Tables

**Table 1:** Measurement model assessment.

Variables	1	2	3	4	5	6	Mean	SD
1. Attitude toward the behavior	1.000	–	–	–	–	–	4.516	1.641
2. Subjective norm	0.395 ^a^ (0.156) ^b^	1.000	–	–	–	–	4.835	1.561
3. Perceived behavioral control	0.409 (0.167)	0.404 (0.163)	1.000	–	–	–	5.149	1.307
4. Perceived knowledge of COVID-19	0.169 (0.029)	0.222 (0.049)	0.268 (0.072)	1.000	–	–	5.544	1.065
5. Psychological risk	0.068 (0.005)	0.134 (0.018)	0.039 (0.002)	0.354 (0.125)	1.000	–	5.642	1.244
6. Behavioral intention for safer destination	0.405 (0.164)	0.478 (0.228)	0.361 (0.130)	0.280 (0.078)	0.221 (0.049)	1.000	4.880	1.390
Composite reliability	0.940	0.879	0.772	0.842	0.809	0.782	–	–
AVE	0.839	0.709	0.533	0.641	0.590	0.546	–	–

Note. Goodness-of-fit statistics for the measurement model: χ^2^ = 189.418, *df* = 117, *p* < 0.001, χ^2^/*df* = 1.619, RMSEA = 0.045, CFI = 0.976, IFI = 0.976, and TLI = 0.968. ^a^ Correlations between the variables are below the diagonal. ^b^ Squared correlations between the variables are within parentheses. AVE: average variance extracted.

**Table 2:** Results of the structural equation modeling (*n* = 305).

Hypothesized Paths	Coefficients	*t*-Values
H1: Attitude toward the behavior → Behavioral intention	0.267	3.775 **
H2: Subjective norm → Behavioral intention	0.349	4.331 **
H3: Perceived behavioral control → Behavioral intention	0.297	3.619 **
H4: Subjective norm → Attitude toward behavior	0.382	5.946 **
H5: Perceived knowledge of COVID-19 → Attitude toward the behavior	0.136	2.048 *
H6: Perceived knowledge of COVID-19 → Subjective norm	0.303	4.277 **
Indirect effect on the behavioral intention:	Total effect on the behavioral intentions:	Explained variance:
β _Subjective norm_ = 0.102 * β _Perceived knowledge of COVID-19_ = 0.173 ** * *p* < 0.05, ** *p* < 0.01	β _Attitude toward the behavior_ = 0.267 ** β _Subjective norm_ = 0.451 ** β _Perceived behavioral control_ = 0.297 ** β _Perceived knowledge of COVID-19_ = 0.173 **	R^2^ (behavioral intentions) = 0.402 R^2^ (attitude) = 0.196 R^2^ (subjective norm) = 0.092

Note. Goodness-of-fit statistics for the structural model: χ^2^ = 195.101, *df* = 80, *p* < 0.001, χ^2^/*df* = 2.439, RMSEA = 0.069, CFI = 0.956, IFI = 0.956, and TLI = 0.942.

**Table 3:** Results of the structural invariance model—intrinsic variety seeking.

Paths	High Group (*n* = 209)		Low Group (*n* = 96)		Baseline Model (Freely Estimated)	Nested Model (Constrained to be Equal)
	β	*t*-Values	β	*t*-Values		
H7a: Subjective norm → Attitude toward the behavior	0.432	5.824 **	0.179	1.507	χ^2^ (170) = 314.748	χ^2^ (171) = 320.400 ^a^
H7b: Attitude toward the behavior → Behavioral intention	0.298	3.514 **	0.173	1.298	χ^2^ (170) = 314.748	χ^2^ (171) = 315.156 ^b^
Chi-square difference test:		Hypotheses testing:		* *p* < 0.05 and ** *p* < 0.01		
^a^ Δχ^2^ (1) = 5.652 and *p* < 0.05 ^b^ Δχ^2^ (1) = 0.408 and *p* > 0.05		H7a: Supported H7b: Not supported ^†^				

**Note.** Goodness-of-fit statistics for the baseline model: χ^2^ = 314.748, *df* = 170, *p* < 0.001, χ^2^/*df* = 1.851, RMSEA = 0.053, CFI = 0.943, IFI = 0.944, and TLI = 0.930. ^†^ While the linkage for the high psychological risk group was significant, the link for the low group was not significant. Hence, although the chi-square difference across the two groups was not significant, the group differences on the attitude and the intention linkages should be meaningfully interpreted.

## Appendix A

**Table A1:** Measurement items for research constructs.

**Attitude toward the behavior** Traveling to a country that is not seriously affected by the COVID-19 outbreak for my next vacation trip is Bad (1)—Good (7). Traveling to a country that is not seriously affected by the COVID-19 outbreak for my next vacation trip is Foolish (1)—Wise (7). Traveling to a country that is not seriously affected by the COVID-19 outbreak for my next vacation trip is Unpleasant (1)—Pleasant (7).
**Subjective norm** Most people who are important to me think I should travel to a country that is not seriously affected by the COVID-19 outbreak for my next vacation trip. Most people who are important to me want me to travel to a country that is not seriously affected by the COVID-19 outbreak for my next vacation trip. People whose opinions I value prefer that I choose a country that is not seriously affected by the COVID-19 outbreak for my next vacation trip.
**Perceived behavioral control** Whether I travel to a tourist destination in a country that is not seriously affected by the COVID-19 outbreak is entirely up to me. I am confident that I can travel to a tourist destination in a country that is not seriously affected by the COVID-19 outbreak if I want to. I have sufficient resources, time, and opportunities to visit a tourist destination in a country that is not seriously affected by the COVID-19 outbreak.
**Perceived knowledge of COVID-19** Compared with the average person, I know the facts about COVID-19. Compared with my friends, I know the facts about COVID-19. Compared with people who travel frequently, I know the facts about COVID-19.
**Psychological Risk** The thought of traveling to tourist destinations in countries seriously affected by the COVID-19 outbreak makes me nervous. The thought of traveling to tourist destinations in countries seriously affected by the COVID-19 outbreak makes me feel psychologically uncomfortable. The thought of traveling to tourist destinations in countries seriously affected by the COVID-19 outbreak causes me to experience unnecessary tension.
**Behavioral intention for safer destinations** I plan to visit a country that is not seriously affected by the COVID-19 outbreak for my next vacation trip after the pandemic has ceased. I will exert effort to travel to a country that is not seriously affected by the COVID-19 outbreak for my next vacation trip after the pandemic has ceased. I am willing to visit a country that is not seriously affected by the COVID-19 outbreak for my next vacation trip after the pandemic has ceased.

Note. All measurement items, except for the items for attitude, were evaluated with a seven-point scale, from “strongly disagree” (1) to “strongly agree” (7).
